# Landfill Leachate and Coagulants Addition Effects on Membrane Bioreactor Mixed Liquor: Filterability, Fouling, and Pollutant Removal

**DOI:** 10.3390/membranes14100212

**Published:** 2024-10-02

**Authors:** Rodrigo Almeria Ragio, Ana Carolina Santana, Eduardo Lucas Subtil

**Affiliations:** Laboratory of Urban Wastewater Treatment and Water Reuse (LabTAUS), Engineering, Modelling and Applied Social Sciences Center, Federal University of ABC, Santo André 09280-560, SP, Brazil; rodrigo.ragio@ufabc.edu.br (R.A.R.); anacarolina0308@hotmail.com (A.C.S.)

**Keywords:** co-treatment, ultrafiltration, coagulation, polyaluminum chloride, tannin-based coagulant

## Abstract

Urban wastewater (UWW) and landfill leachate (LL) co-treatment using membrane bioreactors (MBRs) is a valuable method for managing LL in cities. Coagulants can enhance the filterability of mixed liquor (ML), but the assessment of fouling is still needed. This research aimed to investigate the effects of co-treating synthetic wastewater (SWW) and real LL on an MBR, as well as the impact of adding poly-aluminum chloride (PACl) and Tanfloc SG. Cell-ultrafiltration experiments were conducted with four different feeds: synthetic wastewater, co-treatment with LL (20% *v*/*v*), and co-treatment with the addition of 30 mg L^−1^ coagulants (either PACl or Tanfloc). Co-treatment aggravated flux loss and reduced the recovery rate; however, Tanfloc and PACl improved recovery after cleaning (by 11% and 9%, respectively). Co-treatment also increased cake and irrecoverable/irremovable inorganic resistances, though coagulants reduced the latter, despite a lower fit of the Hermia models during the first hour of filtration. Co-treatment reduced the removal efficiencies of almost all pollutants analyzed, with the most significant impacts observed on the organic fraction. Coagulants, particularly Tanfloc, enhanced overall performance by improving flux recovery and reducing irreversibility, thus benefiting membrane lifespan. In conclusion, Tanfloc addition yielded the best results in terms of filterability and pollutant removal.

## 1. Introduction

One of the most prevalent methods of waste disposal globally is landfilling, which generates a substantial amount of landfill leachate (LL), a highly polluting effluent. The composition and pollutant concentrations of landfill leachate can vary significantly depending on several factors, including the type of deposited material, climatic conditions, soil characteristics, biochemical interactions, disposal time, and operational conditions. These factors influence the chemical and physical properties of the leachate, leading to variations in its composition and pollutant concentrations [1,2,3]. Landfill leachate can be classified into four main categories: the dissolved organic fraction, which includes volatile organic acids and recalcitrant compounds such as humic acids; the inorganic fraction, comprising nitrogen compounds (primarily ammonia), as well as phosphorus, calcium, and magnesium; heavy metals; and xenobiotic organic compounds, such as phenols and pesticides. Each of these categories has specific concentrations and characteristics that can harm the environment. Consequently, landfill leachate is a high-organic load effluent containing recalcitrant materials and humic substances, which presents a significant challenge for effective treatment [4,5,6,7].

A variety of treatment systems were evaluated for LL treatment, including those that have been previously employed for typical domestic effluents (e.g., lagoons, UASB systems, activated sludge, and batch reactors). However, the systems demonstrated limited efficiency for LL treatment, even when only parameters commonly analyzed in sewage were considered (e.g., biochemical oxygen demand (BOD_5_), chemical oxygen demand (COD), and ammonia nitrogen) [8]. In recent decades, the co-treatment of wastewater and landfill leachate (LL) in conventional wastewater treatment plants (WWTPs) has emerged as a potential solution for managing LL. In some cities in emerging countries, this may be the only viable option, given that conventional WWTPs demonstrate higher efficiencies in this context and many advanced systems are not financially feasible [9]. However, this can result in an overload of conventional wastewater treatment plants due to an increase in organic and nitrogenous loads, in addition to the presence of recalcitrant materials. Prior research has demonstrated a detrimental impact on the removal efficiency of activated sludge systems when exposed to LL at concentrations of 10–25% (*v*/*v*) [10,11]. Such processes may have adverse effects on the environment, as WWTPs may fail to meet discharge standards or release heavy metals and xenobiotic compounds that are hazardous even at very low concentrations [12]. Conversely, MBR may offer a solution to mitigate the adverse effects on pollutant removal efficiencies at the co-treatment condition, given the membranes’ high rejection capacity [13]. Previous studies have already demonstrated the efficacy of MBR systems in achieving high organic and nitrogen removal efficiencies, despite the inherent variations in specific LL characteristics (removal from 60 to 90%) and permeate that meet the rigorous effluent release standards associated with co-treatment. These findings have been corroborated by several research studies [2,14,15,16].

A variety of treatment systems have been evaluated for landfill leachate (LL) treatment, including those traditionally used for domestic effluents, such as lagoons, upflow anaerobic sludge blanket (UASB) systems, activated sludge, and batch reactors. However, these systems have shown limited efficiency for LL treatment, even when only parameters commonly analyzed in sewage, such as biochemical oxygen demand (BOD_5_), chemical oxygen demand (COD), and ammonia nitrogen, are considered [8]. In recent decades, co-treating wastewater and landfill leachate (LL) in conventional wastewater treatment plants (WWTPs) has emerged as a potential solution for managing LL. In some cities in emerging countries, this may be the only viable option, as conventional WWTPs typically exhibit higher efficiencies in this context, and many advanced systems are not financially feasible [9]. However, this approach can lead to an overload of conventional wastewater treatment plants due to increased organic and nitrogenous loads, along with the presence of recalcitrant materials. Previous research has shown that activated sludge systems experience a reduction in removal efficiency when exposed to LL concentrations of 10–25% (*v*/*v*) [10,11]. Such processes can have adverse environmental effects, as WWTPs may fail to meet discharge standards or release heavy metals and xenobiotic compounds that are hazardous even at very low concentrations [12]. Conversely, membrane bioreactors (MBRs) may offer a solution to mitigate the adverse effects on pollutant removal efficiency under co-treatment conditions due to the membranes’ high rejection capacity [13]. Previous studies have demonstrated the efficacy of MBR systems in achieving high organic and nitrogen removal efficiencies, despite variations in LL characteristics, with removal rates ranging from 60% to 90%. MBR systems have also been shown to produce permeate that meets the stringent effluent release standards associated with co-treatment [2,14,15,16].

Nevertheless, pollutant removal efficiencies may not be the sole determining factor in the application of MBR systems for co-treatment. It is essential to thoroughly examine the impact of landfill leachate on sludge filterability (i.e., the resistance of the matrix to filtration and its effects on permeate flux) and membrane fouling. Even in low proportions, the presence of hydrophobic compounds, humic and fulvic substances, and other materials could potentially negatively affect both filterability and fouling. While evidence indicates that landfill leachate treatment alone can reduce sludge filterability and intensify membrane fouling in MBR systems [17,18,19], there is currently insufficient evidence to suggest that co-treatment would have the same effects. If co-treatment does indeed exacerbate these issues, it may be possible to mitigate the effects through the use of specific additives, such as commercial coagulants and flocculants, even at low dosages.

One of the most widely used and well-established inorganic coagulants is polyaluminum chloride (PACl). Its direct application in leachate treatment has demonstrated high efficiency in removing pollutants, particularly in terms of color (up to 91.2%) and turbidity (up to 97.7%), and it can also reduce dissolved and colloidal organic matter by up to 36.8% [20]. Research has shown that adding PACl to MBR mixed liquor (ML) at dosages of 0.0025–0.1 gPAC gTSS^−1^ results in a notable increase in membrane flux, ranging from 25% to 58% [21,22,23]. However, aluminum-based coagulants can cause a reduction in pH, necessitating the use of alkalinizing agents to prevent acidification [24]. Additionally, these coagulants have been linked to increased inorganic fouling, which is associated with the deposition of metallic salts and hydroxides on membrane surfaces and within pores [25,26]. Furthermore, residual metallic compounds from these coagulants may pose adverse effects on human health [27] and the environment [28]. The use of natural coagulants, such as tannin-based coagulants, in wastewater treatment can help minimize some of the drawbacks associated with inorganic coagulants. Organic coagulants do not cause issues such as alkalinity consumption and pH reduction [29], and they typically have a reduced environmental impact—especially those derived from plants—compared to their inorganic counterparts. Some studies have even shown that natural coagulants can achieve color, turbidity, and COD removal efficiencies that exceed those of PACl [30,31]. In studies using various organic coagulants, sludge filterability characterization using the time-to-filter method (APHA Method 2710.H) [32] resulted in a 70–90% reduction in time-to-filter, a 233–350% increase in particle size, and an 8–25% improvement in flux [22,33,34,35,36]. Nevertheless, some natural coagulants, such as tannin-based substances (e.g., Tanfloc SG), have yet to be evaluated for their impact on filterability and fouling in leachate, despite their demonstrated efficacy in water and wastewater treatment [37,38]. However, the efficacy of Tanfloc in mitigating fouling has been shown in the treatment of vinasse [39] and UASB reactor effluents [40]. The application of Tanfloc resulted in a reduction in flux loss (final flux without Tanfloc was 30% of the initial, while with 40 mg L^−1^ Tanfloc, it was 55%) and a decrease in fouling resistance (from 3.66 × 10^12^ m^−1^ to 1.09 × 10^12^ m^−1^ with 40 mg L^−1^, in terms of total fouling) [40].

The application of PACl in MBR mixed liquor (ML) has already demonstrated its efficacy in enhancing membrane filterability. However, further evaluation of tannin-based coagulants is necessary, despite the fact that other cationic organic coagulants have shown the ability to improve permeate flux [21,22,23]. Additionally, organic coagulants have the potential to exacerbate irreversible fouling due to residual coagulants and the adhesion of small aggregates to the membrane surface [41,42]. This phenomenon has not yet been investigated in the context of landfill leachate (LL) treatment. Another issue is that the direct addition of coagulants to mixed MBR liquor is typically used for treating domestic or industrial effluents, and this approach is not commonly applied in ML for landfill leachate co-treatment [43,44]. Nonetheless, this combination may prove effective in developing an MBR system that achieves satisfactory performance in LL treatment in urban centers while simultaneously producing high-quality effluent. From this perspective, the objective of this research was to investigate the effects of co-treatment and PACl and Tanfloc additions on sludge filterability, membrane fouling, and pollutant removal efficiencies on MBR mixed liquor ultrafiltration.

## 2. Materials and Methods

### 2.1. Experimental Setup

**Figure 1:**
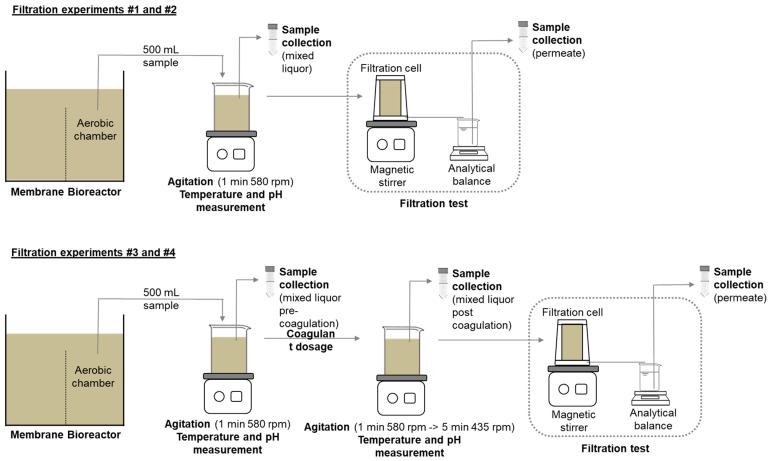
Cell-ultrafiltration experiment design.

To ensure the consistency of experimental conditions and enhance the reproducibility of the results, dead-end filtration experiments were conducted outside of the MBR (Table 1) [45]. Filtration experiments were conducted using an Amicon 8400 stirred filtration cell from Merck/Sigma-Aldrich (St. Louis, MO, USA) (Figure 1) [46,47]. An inert atmosphere was maintained by pressurizing the system with nitrogen gas, and 360 mL of ML samples were stirred at 580 rpm to ensure uniform mixing and shearing on the membrane surface. Due to the concentration effect in the dead-end cell, which may potentially result in higher flux decreases than would be anticipated for the biomass without it, and the observed flux stability at this rate, the experiments were conducted until 33% of the initial volume (120 mL) was collected as permeate. This was then weighed on an analytical balance and converted to volume by water density (998.2 kg m^−3^ at 20 °C). Filtration tests were conducted using a commercial flat sheet ultrafiltration membrane (AMFOR INC. Company, Beijing, China) with a membrane area of 41.8 cm^2^. The membranes had a mean pore size of 22 ± 6 nm and were composed of polyvinylidene fluoride (PVDF), as previously described by Subtil and Mierzwa [48]. Ultrafiltration membranes were selected for their high utility and relatively low cost as a post-treatment option [49]. The initial membrane compaction was conducted for a period of 60 min at a pressure of 1.5 bar. The weight of the permeate was recorded at 30-s intervals (up to the initial 10 min), 60-s intervals (up to minute 60), and 180-s intervals (after 60 min). The experiments were conducted at a room temperature of 22.7 ± 0.7 °C.

To ensure the consistency of experimental conditions and enhance the reproducibility of results, dead-end filtration experiments were conducted outside of the MBR (Table 1) [45]. Filtration experiments utilized an Amicon 8400 stirred filtration cell (Figure 1) [46,47]. An inert atmosphere was maintained by pressurizing the system with nitrogen gas, and 360 mL of mixed liquor (ML) samples were stirred at 580 rpm to ensure uniform mixing and shearing on the membrane surface. Due to the concentration effect in the dead-end cell, which may lead to greater flux reductions than anticipated for the biomass without it, and the observed flux stability at this rate, the experiments were conducted until 33% of the initial volume (120 mL) was collected as permeate. This volume was then weighed on an analytical balance and converted to volume using the water density (998.2 kg m^−3^ at 20 °C). Filtration tests were performed using a commercial flat-sheet ultrafiltration membrane (AMFOR INC.) with an area of 41.8 cm^2^. The membranes had a mean pore size of 22 ± 6 nm and were composed of polyvinylidene fluoride (PVDF), as previously described by Subtil and Mierzwa [48]. Ultrafiltration membranes were chosen for their high utility and relatively low cost as a post-treatment option [49]. Initial membrane compaction was carried out for 60 min at a pressure of 1.5 bar. The weight of the permeate was recorded at 30-s intervals for the first 10 min, 60-s intervals up to minute 60, and 180-s intervals thereafter. The experiments were conducted at a room temperature of 22.7 ± 0.7 °C.

A pilot-scale membrane bioreactor (Figure 1) was operated to collect mixed liquor samples from the aerobic chamber over a three-month period. The operational characteristics of the MBR are detailed in Table S1. Initial microbial activity was provided by aerobic and anaerobic sludge from the Jesus Netto Wastewater Treatment Plant in São Paulo, Brazil. The reactor was operated under two conditions: initially, it was fed solely with synthetic wastewater (sWW) with domestic characteristics; subsequently, it was fed with a mixture of sWW and real landfill leachate (LL) (20% *v*/*v*). The composition of the sWW is presented in Table S2. The methodology followed the approach used by Matsubara et al. [50]. A range of organic nutrients, including proteins, fats, carbohydrates, nitrogen, phosphorus, and trace elements, were used to support biological growth. These conditions led to distinct mixed liquor characteristics, as shown in Table S3.

The landfill leachate used in this research was obtained from the equalization tank at the Santo André Municipal Landfill (Santo André, SP, Brazil), where leachate from two distinct disposal cells—comprising both aged and recent waste—is combined. The characteristics of the collected leachate are presented in Table S4.

The coagulants used in this research were commercially available. Polyaluminum chloride (PACl) was supplied by Purewater© (São Paulo, Brazil), while Tanfloc SG was provided by TANAC© (Montenegro, Brazil). The principal characteristics of these substances are detailed in Table S5. The optimal dosages of both coagulants were determined through Jar Test batch experiments (Figure S2). These tests were conducted at room temperature using 1 L of mixed liquor with rapid mixing (gradient velocity: 180 s^−1^, time: 2 min), slow mixing (20 s^−1^, 15 min), and sedimentation (0 s^−1^, 20 min) [20,21,23,34,51,52,53].

The optimal dosages of the coagulants were determined by measuring dissolved organic carbon, dissolved nitrogen, pH reduction, and color (both apparent and real) [54]. The results are shown in Figure S3. A dosage of 30 mg L^−1^ was chosen for both coagulants, as it lies between the two optimum dosages and allows for a direct comparison under the same conditions.

### 2.2. Fouling Analysis

The filtration tests were based on procedures from previous studies [21,55,56]. Following the compaction process, the initial filtration procedure (Jw0) was conducted using deionized (DI) water. This was followed by filtration of the sample (J), after which physical cleaning was performed, and another DI water filtration (Jw1) was carried out. The procedure was concluded with basic chemical cleaning and a final DI water filtration (J_w2_). Filtration rates were measured as flux (L m^−2^ h^−1^ = LMH).

The membrane cleaning steps were carried out without applying external pressure. Physical cleaning involved manually removing the cake layer formation (removable fouling) using a washing sponge and 100 mL of deionized water for one minute. According to Di Bella and Trapani [57] and other studies [58,59], using the sponge was more effective for removing the superficial cake layer compared to simple washing with DI water. Backwashing was avoided to prevent the removal of both the external cake layer and material adhered to the membrane pores, thus allowing for a more accurate characterization of external fouling [57]. Basic chemical cleaning was achieved by immersing the membrane in a 100 mL solution of sodium hypochlorite (NaOCl) at a concentration of 0.5 g L^−1^ and a pH of 12, adjusted with sodium hydroxide (NaOH). This immersion period was sufficient to achieve a high flux recovery, as supported by previous research [13,21,55].

The flux, normalized flux at 20 °C, permeability, and fouling index (FI30) were estimated according to Equations (1)–(4) [60,61]. Fluxes were calculated as the mean value of the last ten data points for each filtration experiment. All filtration fluxes were normalized to 20 °C and to the initial flux of the respective experiment to facilitate a more accurate comparison of results between samples.

(1) $$J_{i}=\frac{V_{p}}{A\times t}$$

(2) $${Ji}_{T_{0}}=J_{i}\times {\left(\frac{42.5+T_{0}}{42.5+T_{i}}\right)}^{1.5}$$

(3) $$L_{i}=\frac{J_{i}}{TMP}$$

(4) $${FI}_{30}=\frac{J_{30}}{J_{w0}}$$

In this context, J_i_ represents the permeate flux (LMH), V_p_ denotes the permeate volume (L), A stands for the membrane filtration area (m^2^), and t indicates the sampling time (h). J_iT0_ is the permeate flux at the reference temperature (LMH), T_0_ is the reference temperature (°C), and T_i_ is the temperature of the water or mixed liquor during the experiment. LLL represents the permeability (L m^−2^ h^−1^ bar^−1^ = LMH bar^−1^), and TMP is the transmembrane pressure of the experiment (bar).

Additionally, the fouling index (FI30) was determined for filterability analysis as previously described. The index was calculated by dividing the observed flux after 30 min of filtration (J_30_) by the initial water flux (J_w0_) for each filterability test (at the same TMP = 1 bar). The permeate flux data were also used to determine various flux loss ratios (F_Li_), which quantify the flux loss due to total fouling and its constituent fractions, as outlined in Equations (5)–(8) [62,63].

(5) $${FL}_{t}\left(\%\right)=[1-\left(\frac{J}{J_{w0}}\right)]\times 100\;(\mathrm{Total})$$

(6) $${FL}_{r}\left(\%\right)=\left(\frac{J_{w1}-J}{J_{w0}}\right)\times 100\;(\mathrm{Reversible}\;-\;\mathrm{cake}\;\mathrm{layer})$$

(7) $${FL}_{io}\left(\%\right)=\left(\frac{J_{w2}-J_{w1}}{J_{w0}}\right)\times 100\;(\mathrm{Irreversible}\;\mathrm{organic})$$

(8) $${FL}_{ir}\left(\%\right)=\left(\frac{J_{w0}-J_{w2}}{J_{w0}}\right)\times 100\;(\mathrm{Irrecoverable}\;\mathrm{and}\;\mathrm{irreversible}\;\mathrm{inorganic})$$

Flux Recovery Ratios (FRR_i_) were also determined to indicate the antifouling properties of the mixed liquor and to allow for fouling characterization of each sample, according to Equations (9)–(11) [64,65].

(9) $${FRR}_{T}\left(\%\right)=\left(\frac{J_{w2}}{J_{w0}}\right)\times 100\;(\mathrm{Total}\;-\;\mathrm{all}\;\mathrm{cleanings})$$

(10) $${FRR}_{PC}\left(\%\right)=\left(\frac{J_{w1}}{J_{w0}}\right)\times 100\;(\mathrm{Physical}\;\mathrm{cleaning})$$

(11) $${FRR}_{CC}\left(\%\right)={FRR}_{T}-{FRR}_{PC}\;(\mathrm{Basic}\;\mathrm{chemical}\;\mathrm{cleaning})$$

#### Fouling Investigation

The Resistance in Series (RIS) model was applied for fouling characterization according to Equations (12)–(16) for the filtration experiment data [18,21,22,54,55]. In this model, R_i_ represents all filtration resistances (m^−1^), and μ is the permeate viscosity (1.002 × 10^−7^ bar s at 20 °C).

(12) $$R_{T}=R_{m}+R_{f}=R_{m}+({R_{ir}{+R}_{io}+R}_{c})\;(\mathrm{Total})$$

(13) $$R_{m}=\frac{TMP}{\mu \times J_{w0}}\;(\mathrm{Membrane})$$

(14) $$R_{c}=\frac{TMP}{\mu \times J}-(R_{m}+R_{ir}+R_{io})\;(\mathrm{Cake}\;\mathrm{layer})$$

(15) $$R_{io}=\frac{TMP}{\mu \times J_{w1}}-(R_{m}+R_{ir})\;(\mathrm{Irreversible}\;\mathrm{organic}\;\mathrm{fouling})$$

(16) $$R_{ir}=\frac{TMP}{\mu \times J_{w2}}\;(\mathrm{Irreversible}\;\mathrm{inorganic}\;\mathrm{and}\;\mathrm{irrecoverable})$$

The internal inorganic fouling (RII) was not calculated individually due to the adverse effects of acid chemical cleaning on the membranes utilized in this research. Consequently, this fraction was quantified in conjunction with irrecoverable resistance. The investigation of fouling mechanisms was conducted by applying the linearized equations from Hermia models, as presented in Equations (17)–(20) [64,65]. In this context, Q_0_ represents the initial permeate flow (in milliliters per minute), V denotes the cumulative permeate volume (in milliliters), t is time (in minutes), and k is the filtration mass transfer coefficient, whose value is calculated by isolating it in the equations and using the other values obtained in the filtration experiments. Additionally, the regression coefficients (R^2^) were calculated for each curve.

The internal inorganic fouling (RII) was not calculated individually due to the adverse effects of acid chemical cleaning on the membranes used in this research. Therefore, this fraction was quantified together with irrecoverable resistance. Fouling mechanisms were investigated using the linearized equations from Hermia models, as presented in Equations (17)–(20) [64,65]. In this context, Q_0_ represents the initial permeate flow (in mL per minute), V denotes the cumulative permeate volume (in mL), t is time (in minutes), and k is the filtration mass transfer coefficient, which is determined by isolating it in the equations and using the values obtained from the filtration experiments. Additionally, regression coefficients (R^2^) were calculated for each curve.

(17) $$Q=Q_{0}-k_{b}\times V\;(\mathrm{Complete}\;\mathrm{pore}\;\mathrm{blocking})$$

(18) $$\frac{k_{s}\times t}{2}=\frac{t}{V}-\frac{1}{Q_{0}}\;(\mathrm{Standard}\;\mathrm{pore}\;\mathrm{blocking})$$

(19) $$\frac{1}{Q}=\frac{1}{Q_{0}}+\frac{k_{i}}{t}\;(\mathrm{Intermediate}\;\mathrm{pore}\;\mathrm{blocking})$$

(20) $$\frac{k_{c}}{V}=\frac{2\times t}{V}-\frac{2}{Q_{0}}\;(\mathrm{Cake}\;\mathrm{filtration})$$

### 2.3. Analytical Methods

Feed and permeate samples were collected from each filterability test and analyzed for chemical oxygen demand (COD) using method 5220 D, dissolved organic carbon (DOC) using method 5310, UV-absorbing organic compounds (Abs 254) using method 5910 B, total phosphorus (TP) using the Valderrama digestion method [66] followed by 4500 P-E, turbidity using method 2130 B, real color using method 2120 C, and total suspended solids (TSS) using method 2540 D, all in accordance with APHA [32]. The same analyses were performed to characterize the synthetic wastewater (sWW) and landfill leachate (LL) before the experiments. Permeate samples were collected throughout the filtration process. Soluble microbial products (SMP) and extracellular polymeric substances (eEPS) were extracted from the aerobic biomass according to the method described in [67] (Figure S4) and analyzed for proteins [68], carbohydrates [69], and DOC. Mean particle size and particle size distribution were determined by laser beam diffraction using a Mastersizer Hydro 2000G (Malvern Instruments Ltd., Malvern, UK), with an operating range from 0.020 µm to 2000 µm. Particle size distribution was presented as volume-equivalent diameter (%) in the mixed liquor [22].

## 3. Results and Discussion

### 3.1. Co-Treatment Effect

#### 3.1.1. On Mixed Liquor Characteristics

**Figure 2:**
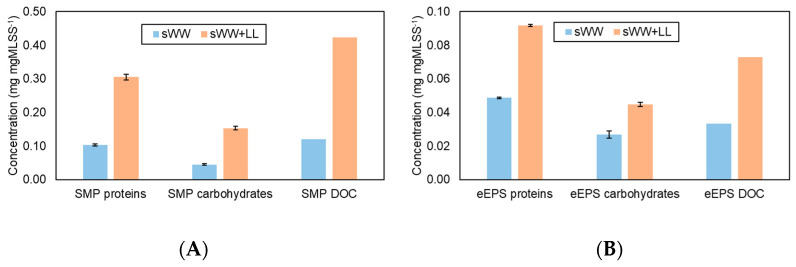
Co-treatment effect on SMP (**A**) and eEPS (**B**) concentrations in aerobic biomass.

The removal efficiency of the SMP and eEPS fractions is illustrated in Figure 2. The data show that co-treatment at 20% *v*/*v* had a significant impact on the increase in SMP. Specifically, the protein concentration increased by 194%, the carbohydrate concentration by 237%, and the DOC fraction by 249%. Similarly, the eEPS concentration rose by 88%, 67%, and 119% for the protein, carbohydrate, and DOC fractions, respectively, compared to the sWW condition. The presence of SMP is often associated with a higher fouling potential, as increases in SMP can reduce membrane filterability [49,70]. Additionally, both EPS fractions (eEPS and SMP) can negatively affect sludge properties. These changes impact the morphology, surface charge, hydrophilicity/hydrophobicity, and adhesive properties of particles, which are influenced by functional groups such as carboxyl, phosphoric, and hydroxyl groups present in proteins and polysaccharides. Particles with these functional groups tend to be negatively charged near neutral pH, increasing particle repulsion and adversely affecting flocculation, stability, adhesion, and dehydration [55,67,71]. The effects of these changes on filterability and membrane fouling will be further investigated; however, it is anticipated that the increased SMP and eEPS concentrations will likely result in reduced filterability and increased fouling.

**Figure 3:**
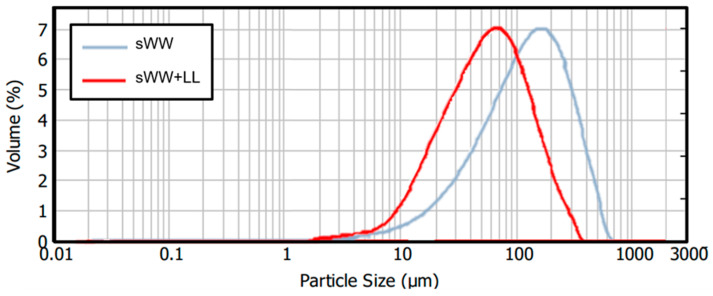
Co-treatment effect on ML particle size distribution.

A 55% reduction in the mean particle size was observed under co-treatment conditions, decreasing from 145.3 µm to 64.8 µm. This reduction can be attributed to the smaller mean particle size of the landfill leachate (11.3 µm), which may impact filterability. Optimal filterability is generally achieved with an average particle size exceeding 80 µm [45]. However, a decrease in membrane flux has been observed with particle sizes below this threshold. Consequently, it can be anticipated that a Harmer effect may occur during filtration under co-treatment conditions, given the observed mean particle size of 64.8 µm. Additionally, changes in particle size distributions were noted, as illustrated in Figure 3. The co-treatment condition resulted in a shift of the distribution curve to the left, indicating an increased frequency of smaller particles. Previous research has suggested that a higher prevalence of small flocs may exacerbate membrane fouling [72,73].

#### 3.1.2. On Fouling

**Figure 4:**
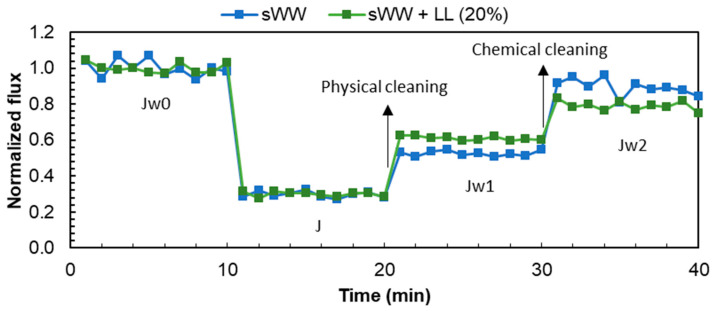
Co-treatment effect on flux loss and recovery by cleaning mechanisms.

The flux data from the filtration experiments are presented in Table 2. The initial flux for the filtration of the samples was observed to be between 14.5 (sWW) and 15.4 (sWW + LL) LMH. While the total flux loss (FL_t_) exhibited a comparable trend in both analyzed conditions, the flux behavior with co-treatment (Figure 4) and the FL and FRR types (Table 2) displayed notable discrepancies. The co-treatment resulted in an increase in reversible fouling, as evidenced by the observed increases in FL_r_ and FRR_PC_, from 19 to 29% and from 53 to 61%, respectively. Furthermore, the flux loss due to irrecoverable and irreversible inorganic fouling also increased from 11 to 21%, which may justify the 10% reduction in FRR_T_ at co-treatment conditions. These alterations in filterability coincided with a reduction in FL and FRR due to irreversible organic fouling. The fouling index (FI30) exhibited a decline from 0.45 to 0.34 under co-treatment conditions. This indicates that the co-treatment condition exhibited a diminished proportional flux after the same filtration period (30 min), thereby reinforcing the notion that co-treatment impairs flux behavior.

The flux data from the filtration experiments are presented in Table 2. The initial flux for the filtration of samples ranged from 14.5 LMH (for sWW) to 15.4 LMH (for sWW + LL). While the total flux loss (FL_t_) showed a similar trend under both conditions, there were notable differences in flux behavior with co-treatment (Figure 4) and the flux loss (FL) and flux recovery ratios (FRR) types (Table 2). Co-treatment led to an increase in reversible fouling, as evidenced by the increases in reversible flux loss (FL_r_) and the flux recovery ratio with post-cleaning conditions (FRR_PC_), which rose from 19% to 29% and from 53% to 61%, respectively. Moreover, flux loss due to irrecoverable and irreversible inorganic fouling increased from 11% to 21%, which helps explain the 10% reduction in the total flux recovery ratio (FRR_T_) under co-treatment conditions. These changes in filterability were accompanied by a reduction in FL and FRR due to irreversible organic fouling. The fouling index (FI30) decreased from 0.45 to 0.34 under co-treatment conditions, indicating a lower proportional flux after 30 min of filtration. This reinforces the notion that co-treatment impairs flux behavior.

**Figure 5:**
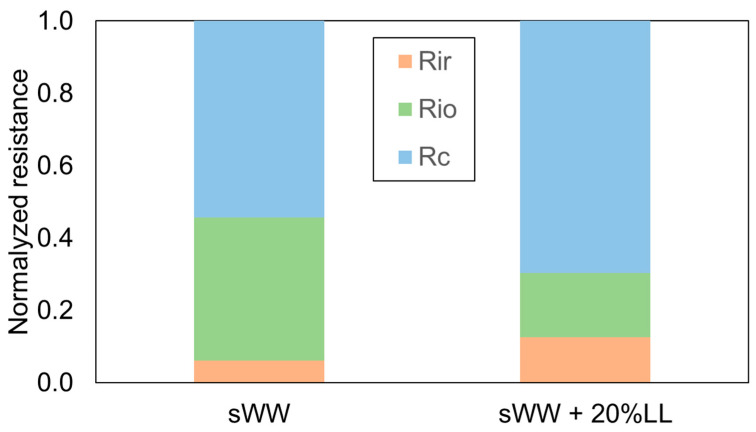
Co-treatment effect on R_f_ fractions.

These findings are further supported by the data presented in Figure 5. The increased occurrence of reversible fouling under co-treatment conditions was indicative of a subsequent rise in permeate flux following the implementation of a physical cleaning process. Conversely, the lower total flux recovery ratio (FRR) with landfill leachate addition indicated a reduction in water flux following all cleaning procedures. These results suggest that the use of synthetic effluent resulted in a mixed liquor that was more effectively cleaned compared to the co-treatment condition with real effluent (landfill leachate). This observation can be attributed to the increase in soluble microbial products (SMP) and extracellular polymeric substances (EPS) values, as well as the reduction in particle size with the addition of landfill leachate.

Prior research has demonstrated that an ultrafiltration membrane bioreactor (MBR) fed with synthetic wastewater (sWW) exhibits higher removable fouling compared to when fed with real wastewater (rWW). The absence of humic-like substances, commonly found in landfill leachate, and the presence of proteins with higher molecular weights (>600 kDa) were identified as the primary distinguishing factors between synthetic and real wastewater [74].

The higher flux recovery ratio (FRR) due to physical cleaning (61%) under co-treatment conditions, compared to the sWW condition (53%), can be attributed to the significant diversity of molecules and compounds present, which may interact with each other and accumulate on the cake filtration layer. This accumulation can be more effectively removed through physical cleaning. For example, an increase in small particles could fill gaps in the cake layer, potentially making it more resistant to cleaning.

The lower total FRR observed under co-treatment conditions is consistent with the increased concentration of soluble microbial products (SMP) previously discussed (Figure 2A). Elevated SMP concentrations are known to correlate with reduced filterability of mixed liquor and increased fouling potential [49,60,70].

The participation of total resistance in fouling showed a similar trend under both conditions (Table 3), although it was numerically lower during co-treatment. This may be attributed to the substantial reduction in mixed liquor suspended solids (MLSS) concentration, from 2.50 to 1.43 gTSS L^−1^, observed under co-treatment conditions. Previous studies have reported a correlation between lower MLSS concentrations and reduced fouling. This relationship is supported by a nearly linear correlation between membrane surface solid deposition and MLSS concentration [75,76,77]. A higher sludge concentration could lead to a more rapid formation of the sludge cake and, consequently, a more rapid development of fouling [78].

Despite the numerical discrepancy, the proportions of fouling resistances displayed a significant divergence. In both conditions, the cake layer resistance remained the dominant component of the total fouling resistance, a finding consistent with previous studies on submerged MBRs treating domestic wastewater [18,79]. Under co-treatment conditions, the cake layer resistance increased from 54% to 70% (Figure 5). This evidence suggests that the greater diversity of compounds in co-treatment conditions leads to more complex interactions among them, resulting in increased aggregation. This aggregation forms a thicker cake layer that is more amenable to removal through physical cleaning.

The reduced effectiveness of conventional chemical cleaning under co-treatment conditions aligns with the observed decrease in internal organic fouling resistance, which dropped from 40% to 18% (Figure 5). However, this condition led to a doubling of irrecoverable and irreversible inorganic fouling resistance, increasing from 6% to 13%. This increase is likely responsible for the previously observed reduction in the total Flux Recovery Ratio (FRR) (Table 2).

The application of the Hermia model provides a deeper understanding of the fouling mechanisms, with the results summarized in Table 4. As shown, intermediate blocking (IB), standard blocking (SB), and cake layer filtration (CF) were the predominant fouling mechanisms during the initial two hours of filtration under both synthetic and co-treatment conditions. This is reflected by the highest linear correlation coefficients, with cake layer filtration also showing a significant correlation. Complete blocking occurred less frequently than the other three mechanisms.

The coexistence of multiple fouling mechanisms is frequently referenced in the literature as a phenomenon that typically manifests at the beginning of filtration. Multiple fouling mechanisms can occur simultaneously on the membrane surface, with no single mechanism clearly dominating [64,80]. Several studies have observed this phenomenon during the early stages of dead-end ultrafiltration. However, they also indicated that the cake filtration mechanism tends to predominate at later stages. This was not confirmed in our initial analyses because the linear square fitting was very close [75,81,82].

A higher degree of fitting was observed during the first 30 min of filtration with synthetic wastewater compared to the remaining filtration time. In contrast, co-treatment conditions exhibited a higher correlation during the second hour of filtration (60–120 min). Between 30 and 60 min, the linear correlation coefficients showed a notable decline in both conditions, with a more pronounced decrease observed under co-treatment conditions.

The reduced correlation between fouling mechanism models under co-treatment conditions can be attributed to the increased complexity and diversity of interactions between the mixed liquor and the membrane due to the addition of landfill leachate. As filtration progressed, the fouling mechanisms became clearer, as evidenced by the increased correlation coefficients observed during the later stages (60–120 min).

The unique pattern observed in the linear correlation coefficients of Hermia models under co-treatment conditions may be related to the higher concentrations of carbohydrates and proteins, especially in their soluble forms. Previous research has shown that ultrafiltration (UF) systems foul more rapidly at elevated soluble microbial product (SMP) concentrations, either through pore blocking (due to the small size of protein particles relative to membrane pores) or cake layer formation. Larger carbohydrate molecules, or their interaction with proteins as biopolymer cluster (BPC) precursors, can exacerbate cake layer formation [49,56,83,84]. The increase in small particles in co-treatment conditions may also hinder pore-blocking mechanisms, leading to reduced accuracy in model fitting. These findings are linked to increased fouling irreversibility, which is often associated with pore blocking and external fouling, manifesting as an accumulation of a cake layer. Consequently, this suggests the need for more frequent membrane cleaning and potentially a shorter membrane lifespan in membrane bioreactors (MBRs) under co-treatment conditions.

#### 3.1.3. On Pollutant Removal

**Figure 6:**
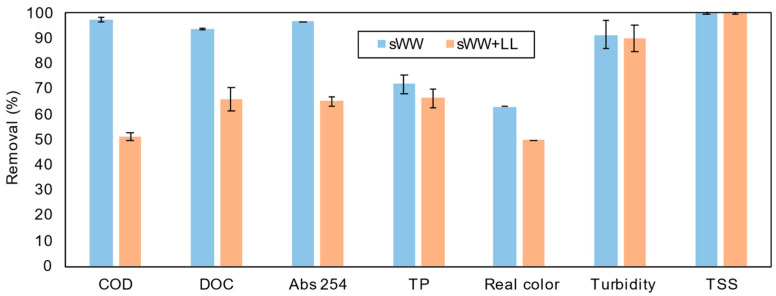
Cotreatment effect on pollutants removal efficiency.

The effect of co-treatment on pollutant removal was measured, and the results are presented in Figure 6. Co-treatment reduced the removal efficiencies for all pollutants except total suspended solids (TSS), which is typical for a membrane bioreactor (MBR) system. The most significant reductions were observed for chemical oxygen demand (COD), dissolved organic carbon (DOC), and absorbance at 254 nm.

The COD removal efficiency for synthetic wastewater (sWW) was nearly 100%, with influent concentrations of 490 ± 39 mg L^−1^. However, co-treatment with landfill leachate (LL) reduced the removal efficiency to 51 ± 3.9%, with higher influent and effluent concentrations (656 ± 210 mg L^−1^ and 321 ± 12 mg L^−1^, respectively). An increase in both influent and effluent concentrations was also observed by Çeçen et al. [10] when co-treating with LL at a concentration of 4–25% (*v*/*v*). This reduction in COD removal with co-treatment was expected, as leachate introduces compounds that are more challenging to degrade, including refractory and humic substances [7,65]. However, the observed COD removal rate was lower than that reported in the literature for co-treatment in membrane systems. For instance, COD removal rates exceeding 90% have been achieved with elevated LL concentrations (3–40% *v*/*v*) [15]. The authors noted that LL concentrations should not exceed 10% (*v*/*v*) due to more pronounced effects at higher concentrations. Moreover, higher COD removals (60–80%) have been reported in other systems without membranes, using 2–20% *v*/*v* LL co-treatment [8,85].

Co-treatment conditions also resulted in a reduction in DOC removal efficiency, from 93.7% to 66.0%. Çeçen et al. [10] observed a higher DOC removal rate (80%) with a lower LL ratio (2–5% *v*/*v*). These findings suggest that the inclusion of leachate introduces recalcitrant organic compounds [16]. In an enhanced membrane bioreactor (EMBR) with 20% *v*/*v* LL co-treatment, COD removal was 86 ± 5% (204 mg L^−1^ residual), and BOD_5_ removal was 98 ± 1% (10 mg L^−1^ residual). This indicates the significant presence of recalcitrant organic compounds in the leachate, which may potentially permeate the membrane.

The increase in recalcitrant compounds was confirmed through Abs 254 measurements, which showed a significant rise from 2250 cm^−1^ to 5360 cm^−1^ with the addition of landfill leachate (LL). This analysis quantifies humic and fulvic substances, which are more resistant to degradation. Consequently, the removal efficiency decreased from 96.7% to 65.0%, and the Abs 254 of the permeate increased from 0.075 cm^−1^ to 0.990 cm^−1^ under co-treatment conditions. Additionally, Abs 254 removal was limited to only 13 ± 1% in an enhanced membrane bioreactor (EMBR) with 20% *v*/*v* LL co-treatment with synthetic wastewater (sWW). The low removal efficiency was attributed to a combination of factors, including a relatively short hydraulic retention time (HRT) and the presence of recalcitrant compounds in the leachate, which require a longer time to degrade.

The reduced Abs 254 removal efficiency is linked to the decline in real color removal efficiency. This parameter dropped notably from 63.1% to 49.9% under co-treatment conditions. Previous studies have observed similar limitations on real color removal due to restricted humic and fulvic acid removal [86]. Figure S3 illustrates the change in real color. The mixed liquor’s real color under co-treatment conditions was 3788 ± 1185 uC, and it decreased to 1898 uC in the permeate, which is still relatively high. Despite this intermediate reduction, the permeate real color remains elevated, necessitating additional post-treatment.

The impact of co-treatment on turbidity removal was minimal, decreasing from 91.4% to 89.8%. Çeçen et al. [10] achieved 83% turbidity removal with 20% LL co-treatment, as shown in Table 3. This result was obtained in a different system. These findings demonstrate the high efficiency and stability of the membrane system, even under co-treatment conditions. Furthermore, high total suspended solids (TSS) removal was achieved for both conditions, exceeding 99%, which aligns with expectations for membrane bioreactor (MBR) ultrafiltration systems.

### 3.2. Coagulants Effect

#### 3.2.1. On Mixed Liquor Characteristics

The addition of Tanfloc did not result in a significant change in the pH of the mixed liquor, which slightly decreased from 7.64 to 7.49. However, the addition of PACl caused a more substantial reduction, from 6.96 to 5.65. To prevent the low pH from affecting the filterability tests and to maintain consistent conditions, the pH was adjusted to 7.00 with a NaOH solution immediately before starting the tests. As expected, the aluminum coagulant had a noticeable impact on pH [23].

**Figure 7:**
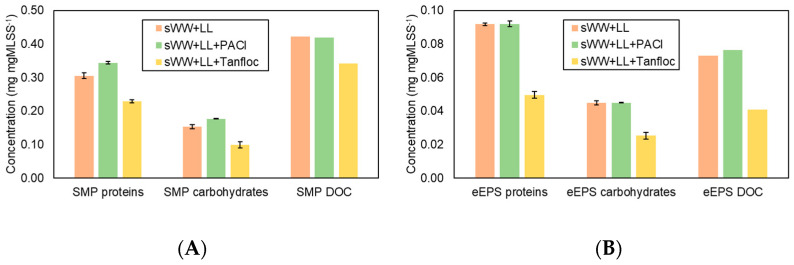
Coagulants effect on SMP (**A**) and eEPS (**B**) concentrations in aerobic biomass.

Figure 7 illustrates the impact of the coagulants on the soluble microbial products (SMP) and extracellular polymeric substances (eEPS) fractions. The addition of PACl did not reduce the SMP and eEPS fractions. The observed increases may be attributed to system instabilities following each sample collection, or the dosage might have been insufficient for effective coagulation/flocculation. In contrast, Tanfloc was effective in reducing all SMP fractions by 19–35% and eEPS concentrations, with more pronounced reductions in eEPS fractions (46% for proteins and 44% for carbohydrates and dissolved organic carbon (DOC).

Prior research has demonstrated the effectiveness of using various organic coagulants for the removal of soluble microbial products (SMP), with observed reductions ranging from 10% to 70% [22,30,32,87]. Additionally, reducing SMP has been shown to be crucial in enhancing filterability and mitigating fouling [49,56,70]. Based on these findings, it is anticipated that the addition of Tanfloc will increase the flux recovery ratio (FRR) and cake layer resistance, leading to more effective removal of fouling. It is known that extracellular polymeric substance (eEPS) concentration correlates with the viscosity of the mixed liquor and is also linked to the rate of membrane fouling [75]. Therefore, a reduction in eEPS due to the addition of Tanfloc is expected to lead to decreased fouling.

The mean particle size improved by 124% and 23% with the addition of PACl and Tanfloc, respectively. The mean particle size increased from 64.8 µm in the co-treatment condition to 145.1 µm (comparable to the synthetic wastewater condition) with PACl and to 79.5 µm with Tanfloc. The results with PACl are significantly better than the threshold indicated in other studies [45], above which good filterability is observed. However, the impact of Tanfloc on filterability remains uncertain based on these results.

Prior research has documented a significant increase in average particle size with the use of PACl and other aluminum and inorganic coagulants. For example, one study reported a maximum particle size of 160 µm with a dosage of 0.1 mM Al^3+^, representing a 167% increase from 60 µm [88]. Another study observed a 260% enhancement in mean particle size with a dosage of 20 mg L^−1^ PACl, increasing the size from 125 nm to 488 nm [22]. This enhancement was attributed to the optimal neutralization of the surface charge of sludge particles, leading to the aggregation of neutralized sludge flocs. It was noted that dosages of Al^3+^ salts below or above the optimal range resulted in reduced particle size values [22,89].

In comparison, the mean particle size with Tanfloc showed a slight increase of 23% (from 64.8 µm to 79.5 µm), which is less significant than the improvements reported with PACl. These findings are inconsistent with the existing literature. For instance, Ragio et al. [40] observed a reduction in particles smaller than 40 µm in UASB effluent (from 88.1% to 57.7%) and an increase in particles larger than 200 µm (from less than 1% to 11.7%) following the addition of 40 mg L^−1^ of the Tanfloc active principle. Zhang et al. [90] reported a considerable improvement of 115% (from 55 µm to 118 µm) with the addition of 0.7 g gDS^−1^ of polydimethyldiallylammonium chloride (PDMDAAC), another organic coagulant. Chitosan, an organic and cationic coagulant, achieved the greatest improvement, with a 300% increase in mean particle size (from 110 nm to 440 nm) at a dosage of 10 mg L^−1^ [22]. Another experiment with chitosan resulted in an increase in mean particle size from 125 µm to 151 µm and further to 258 µm with a dosage of 200 mg L^−1^.

Prior research has shown that deviations from the optimal dosage range can reduce the average particle size. This reduction is often due to the adsorption of polymers, which increases electrostatic repulsion [90]. This phenomenon may have occurred in the present study, potentially explaining the limited improvement in mean particle size observed with the addition of Tanfloc. Further research could provide a more comprehensive investigation of this parameter at varying dosages.

**Figure 8:**
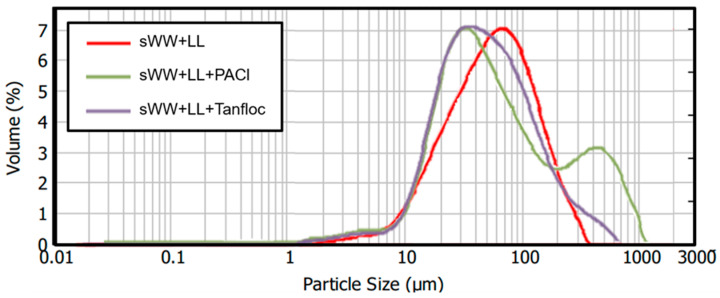
Coagulants effect on ML particle size distribution.

The analysis of particle size distribution revealed an intriguing pattern (Figure 8). The addition of PACl resulted in a two-peak curve, with one peak situated below and one above the peak observed in the co-treatment condition curve. This indicates an increase in the number of flocs with larger sizes (200–1000 µm) when PACl was added. However, it also suggests that PACl addition led to the formation of smaller particles (15–45 µm) that did not aggregate into larger flocs and may persist in the mixed liquor. These effects could be attributed to underdosing. As a result, the floc structure became less compact and exhibited a lower effective density compared to that formed with a higher dosage. This suggests that further filterability tests with additional PACl dosages might be needed to achieve more optimal results in terms of particle size. Similar effects were observed with Tanfloc, although they were less pronounced. The addition of Tanfloc improved the distribution of particle sizes, reducing the proportion of particles in the 15–50 µm range and increasing the proportion in the 300–600 µm range. However, the proportion of particles in the higher range remains lower than that observed with PACl. Therefore, evaluating additional dosages of Tanfloc may help determine the optimal concentration.

The addition of PAC resulted in a significant increase in particle size, which might be expected to improve filterability and reduce fouling. However, larger particle sizes do not always correlate with optimal filterability improvements when using flux enhancers in mixed liquor [91]. Additionally, the impact of large flocs on flux and fouling can vary depending on hydrodynamic conditions [92]. These particles could potentially deposit on the membrane and act as foulants, thereby reducing permeate flux. Conversely, they could function as “moving filters”, trapping soluble and colloidal substances and thereby alleviating fouling and enhancing permeate flux. The use of aluminum-based coagulants may lead to increased irreversible fouling, potentially resulting in a dense cake layer and the crystallization of aggregate particles [93]. Moreover, a reduction in particle size might be associated with increased fouling mitigation, especially under low transmembrane pressure (TMP) conditions [76]. This controversial effect will be further investigated in the following sections.

#### 3.2.2. On Fouling

The initial fluxes with coagulants were 12.9 LMH for Tanfloc and 14.8 LMH for PACl. The FI30 calculations showed an increase from 0.34 to 0.38 with PACl addition and a decrease to 0.31 with Tanfloc addition. These changes indicate that the mixed liquor (ML) with PACl exhibited a higher proportional flux after 30 min of filtration. In contrast, the ML with Tanfloc showed a lower proportional flux after the same filtration time, even lower than the flux observed with the co-treatment condition without coagulants. As confirmed by the FL_t_ calculation (Table 5), PACl resulted in a similar flux loss compared to co-treatment without coagulants (71% and 70%, respectively). However, the total fouling resistance ratio (FRR) increased from 79% to 86% with PACl addition due to a reduction in irreversible fouling (FL_ir_ decreased from 21% to 14%) (Table 5). Calculations of fouling resistance (FL) and FRR fractions also showed an increase in reversible fouling (cake layer; FL_r_ and FRR_PC_) and a reduction in irreversible organic fouling (internal; FL_io_ and FRR_CC_).

The addition of Tanfloc resulted in an increase in total fouling loss (FL) from 70% to 75%. Concurrently, there was an improvement in the total fouling resistance ratio (FRR), increasing from 79% to 88%. This observed increase in FRR can be attributed to a rise in internal organic fouling, as shown in Table 5. Specifically, Tanfloc led to a notable increase in both internal fouling loss (FL_io_) and internal organic fouling resistance (FRR_CC_), rising from 18% to 30%. Additionally, the addition of Tanfloc resulted in a reduction in irreversible fouling loss (FL_ir_) from 21% to 12%, a more pronounced effect compared to PACl.

**Figure 9:**
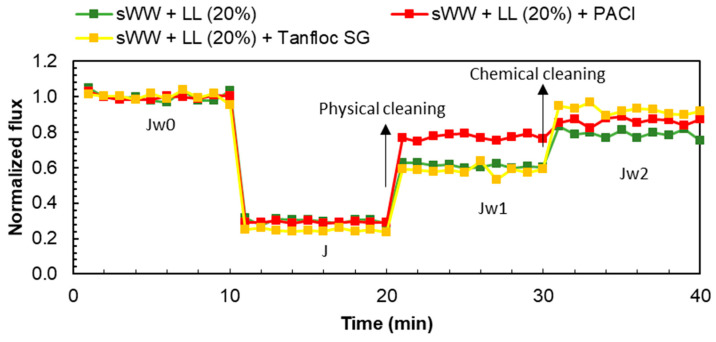
Coagulants effect on flux loss and recovery by cleaning mechanisms.

In summary, the addition of PACl modified the fouling characteristics to enhance reversibility, thereby increasing the impact of the cake layer, which can be more easily removed through physical cleaning (Figure 9). Conversely, the addition of Tanfloc led to an increase in irreversible but recoverable fouling (organic internal fouling), which can be addressed through basic chemical cleaning (Figure 9).

The effects of the coagulants are related to induced aggregation and improvements in particle size, as discussed earlier. PACl resulted in a larger mean particle size (145.1 µm) compared to Tanfloc (79.5 µm). Previous research has shown that larger particles are better retained on the cake layer, which facilitates removal through physical cleaning [92]. The smaller mean particle size observed with Tanfloc indicates that smaller particles or flocs are more likely to be trapped within membrane pores rather than being retained on the membrane surface or cake layer [94].

The application of PACl did not result in a reduction in soluble microbial products (SMP) and extracellular polymeric substances (eEPS) concentrations (Figure 7). However, a qualitative change in these fractions may have occurred, potentially enhancing interactions between them and promoting cake layer formation. This could explain the observed increase in reversible fouling loss (FL_r_) and reversible organic fouling resistance (FRR_PC_). In contrast, Tanfloc reduced both SMP and eEPS concentrations (Figure 7), leading to increased internal fouling loss (FL_io_) and internal organic fouling resistance (FRR_CC_). This indicates that the flux loss with Tanfloc was less due to the cake layer and more because of internal organic fouling. Therefore, Tanfloc’s impact on the mixed liquor was more quantitative, while PACl’s effects led to more qualitative changes, such as alterations in surface floc charge or size. As previously discussed (Figure 9), increasing PACl dosage significantly impacted particle size.

Previous studies have shown that the addition of coagulants, especially PACl and ferric chloride, can reduce fouling resistance [52,95]. However, in our study, fouling resistance (R_f_) increased with the addition of both PACl and Tanfloc, as shown in Table 6. This increase cannot be explained solely by the relatively low coagulant dosages used in this research compared to previous studies (which often used dosages greater than 50 mg L^−1^). Consequently, the beneficial effect observed in this study is related to the reversibility of fouling rather than a reduction in overall fouling resistance.

**Figure 10:**
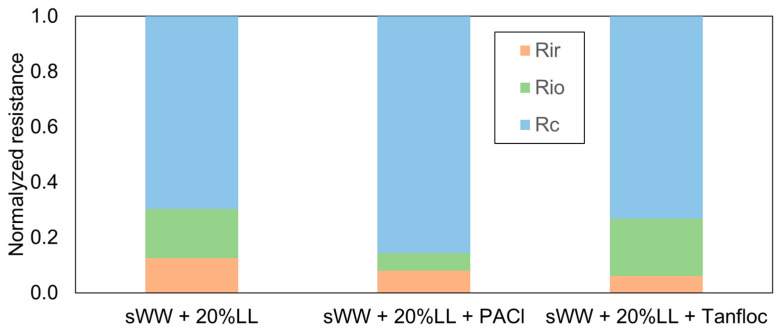
Coagulants effect on R_f_ fractions.

Furthermore, the addition of PACl led to a 23% improvement in cake layer resistance, while Tanfloc resulted in only a 4% improvement in the same fraction (Figure 10). Additionally, Tanfloc caused a 17% increase in internal organic resistance, whereas PACl and Tanfloc both led to reductions of 38% and 54% in irrecoverable and internal inorganic resistance, respectively (Table 6). These effects, related to filterability analysis, indicate improved fouling removal through physical and basic chemical cleaning and a likely increase in the flux recovery rate. These findings are supported by the filterability results presented earlier in Table 5.

It has been previously observed that the cake layer resistance relative to total fouling resistance increases with the addition of coagulants [22,55]. This observation is consistent with findings from other studies, which have also noted a greater contribution of cake layer resistance to total fouling resistance across various conditions (with and without coagulants) [22,55]. For example, the addition of PACl (20 mg L^−1^) and chitosan resulted in increases in cake resistance of 10% and 20%, respectively [31].

The observed enhancement in internal and organic fouling resistance (from 18% to 21%) associated with Tanfloc addition aligns with the improvement in flux recovery rate (FRR) resulting from basic chemical cleaning (from 18% to 30%) (Table 5). This finding supports the hypothesis that, despite its smaller particle size, Tanfloc leads to a greater degree of organic fouling compared to the co-treatment condition. This phenomenon may be attributed to the coagulant itself acting as a foulant, where some residue does not interact effectively with existing particles or forms very small flocs [41,42]. Both PACl and Tanfloc were observed to reduce irrecoverable and internal inorganic fouling (Table 6), although the effect was more pronounced with Tanfloc. Prior research has documented reductions in irrecoverable fouling resistance with other inorganic coagulants, such as PACl and FeCl3 [53,95]. However, the superior performance of the organic coagulant, Tanfloc, compared to inorganic coagulants represents a significant finding in the co-treatment of landfill leachate (LL). This may be attributed to Tanfloc’s organic nature. Further studies are needed to confirm this relationship between coagulant composition and its effect on irrecoverable and inorganic fouling.

It has been demonstrated that the addition of PACl has a beneficial effect on mitigating fouling and improving filterability, as evidenced by increases in FI30 and FRR when used with other metallic coagulants, such as polymeric ferric sulfate [96] and ferric chloride [70]. However, a reduction in SMP and eEPS concentrations has not been consistently observed. This supports the controversial and unclear effects of SMP and eEPS reported in both this research and the literature [45,70,75,78,96,97]. The current findings may be related to changes in the characteristics of the mixed liquor, which can result from the addition of coagulants [91]. In contrast, the addition of Tanfloc led to a reduction in SMP and eEPS, along with improvements in FRR and decreases in both irrecoverable and internal inorganic fouling. However, the effect of Tanfloc on FI30 remains controversial.

When PACl was added during co-treatment, a better fit was observed between the flux results and the applied models throughout all filtration times compared to co-treatment without coagulants (Table 7). This improvement is advantageous because it enhances the identification of fouling mechanisms involved in filtration, facilitating the prediction and prevention of flux loss [64,98].

The high fit of the cake filtration model, especially during the initial filtration period, corroborated the filterability and RIS model outcomes. The results showed a higher FRR due to physical cleaning and a greater contribution of cake layer resistance to the total fouling resistance compared to the co-treatment condition. The dominance of the cake filtration model from the beginning indicates the formation of a thick gel layer on the membrane surface [99]. However, PACl addition demonstrated the most accurate fit to the complete blocking model after the initial thirty minutes of filtration. This suggests that the earlier fouling mechanisms, particularly cake formation, were naturally removed during filtration and replaced by complete pore blocking.

The application of Hermia models to experiments with Tanfloc did not yield a correlation with experimental data in the first 30 min of filtration. This limitation hindered the understanding of the fouling mechanisms involved at the outset. Such behavior could be attributed to an unidentified mechanism or a combination of several mechanisms [80]. Alternatively, it is possible that the matrix influence is responsible for this phenomenon, as [39] found a good fit for the cake layer and intermediate blocking during vinasse microfiltration with a tannin-based coagulant aid.

Nevertheless, the coefficients showed an increase with filtration time. After 60 min of experimentation, it was confirmed that all fouling mechanisms were occurring, particularly intermediate blocking (IB), standard blocking (SB), and cake formation (CF). The improvement in CF after the first 60 min of Tanfloc filtration and the reduction in this parameter after the first 60 min of PACl filtration suggest that a cake layer forms more rapidly with PACl addition. In contrast, the formation of a cake with Tanfloc allows for two additional observations: first, a greater deposition of smaller organic particles within the membrane, contributing to a higher FRR due to basic cake layer resistance (CC); second, a larger proportion of internal organic fouling in the total fouling resistance.

#### 3.2.3. On Pollutant Removal

**Figure 11:**
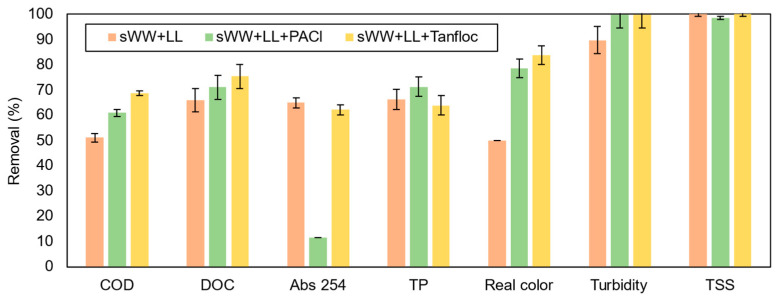
Coagulants effect on pollutants removal efficiency.

As shown in Figure 11, the addition of coagulants improved the removal efficiency of nearly all pollutants. Specifically, PACl and Tanfloc both enhanced COD removal, reducing its concentration in the permeate to 256 ± 9 mg L^−1^ and 205 ± 5 mg L^−1^, respectively.

The COD removal rates of 61.0 ± 1.3% and 68.7 ± 0.8% achieved with coagulants were comparable to, and even exceeded, the results of previous studies examining the impact of PACl on MBR systems without co-treatment and with higher coagulant dosages (200–1900 mg L^−1^) [21,23]. Additionally, a significantly lower coagulant concentration (30 mg L^−1^) in the co-treatment condition achieved a COD removal comparable to the maximum observed in the literature (26–61.6%) for higher dosages (650–6000 mg L^−1^) of landfill leachate [20,100,101].

A similar outcome was observed with Tanfloc addition. The removal efficiency obtained (68.7 ± 0.8%) was in line with the higher efficiencies reported in the literature (32–69%) for pure landfill leachate, despite using a much lower dosage (30 mg L^−1^) compared to those typically employed (100–1100 mg L^−1^) [29,30,101,102]. These findings indicate that comparable removal efficiencies can be achieved with these coagulants at lower dosages (30 mg L^−1^) under co-treatment conditions.

It is noteworthy that these results were achieved through the direct addition of coagulants to the MBR mixed liquor, a methodology that differs from the conventional approach, which typically involves coagulation–flocculation prior to membrane filtration. This direct approach yielded satisfactory results while simplifying the treatment process.

Moreover, the addition of PACl and Tanfloc resulted in increased DOC removal, reaching 71.1 ± 4.7% and 75.4 ± 4.7%, respectively. Although these indices are lower than those achieved with PACl addition to MBR systems (84.6–92.3%) without leachate co-treatment [35], the results are still notable. For direct leachate treatment, comparable removal efficiency (78%) was observed with ferric chloride (FeCl_3_) at a higher dosage (3 g L^−1^) and lower pH (4) in a previous study [51]. Thus, the addition of PACl and even Tanfloc to the co-treatment process at lower dosages demonstrated favorable outcomes in terms of organic carbon removal compared to traditional non-polymerized coagulants.The total phosphorus (TP) removal rate was 66.3% in the absence of a coagulant and 63.9% with Tanfloc. In contrast, the addition of PACl improved TP removal efficiency to 71.3%, comparable to the 71.9% observed in synthetic wastewater conditions. This performance of PACl aligns with expectations, given the preliminary tests and previous studies that reported phosphorus removal rates as high as 90% with aluminum coagulants [23,103,104]. However, the phosphorus concentration in the permeate (5.4 mg P L^−1^) was significantly higher than that achieved by [14] in co-treatment with an MBR. Further investigation is needed to determine whether extended testing and specific conditions could improve phosphorus removal outcomes. Organic coagulants, such as Tanfloc, have been shown to be less effective in phosphorus removal due to the high negative charge of phosphate ions (−3) and mono-hydrogen phosphate (HPO_4_^2−^), which require substantial electrostatic force for destabilization. This level of destabilization is typically achieved with aluminum-based coagulants [105].

Despite these observations, PACl’s performance in total phosphorus removal did not translate to an improvement in Abs 254 nm reduction. PACl resulted in a significant drop in removal efficiency from 65.0% to only 11.7%, while Tanfloc had a negligible effect on Abs 254 nm, with a removal efficiency of 62.2%. The Abs 254 nm parameter is not commonly used to evaluate PACl’s effectiveness in leachate treatment or MBR systems. Previous studies involving organic coagulants, particularly Tanfloc variants (SH and SG), have shown Abs 254 nm removal efficiencies ranging from 9.95% to 59.3% with dosages of 1.0 to 1.5 mg L^−1^ for leachate treatment [102,106,107]. In this study, a higher removal efficiency (62.2%) was achieved with a co-treatment condition and a much lower dosage (30 mg L^−1^), likely due to leachate dilution and the presence of the coagulant. It is also noteworthy that previous research has identified humic and fulvic acids, measured by Abs 254 nm, as the primary organics remaining after coagulation of leachate, which may explain the low removal efficiency for this parameter in this study.

The addition of coagulants significantly improved color removal, with efficiencies increasing from 49.9% in the co-treatment condition to 78.5% with PACl and 83.7% with Tanfloc SG. These results are comparable to and even exceed those reported in previous studies that investigated the direct application of PACl (72–96.8%) and Tanfloc (27.8–88%) for landfill leachate treatment [30,100,101,106,107]. The effectiveness of coagulants in this study was achieved with a much lower dosage of 30 mg L^−1^ compared to dosages used in prior research, which ranged from 650 to 6000 mg L^−1^ for PACl and 100 to 1500 mg L^−1^ for Tanfloc. For turbidity removal, both coagulants improved their efficiency from 89.8% to nearly 100%, with residual turbidity falling below 0.1 NTU. This performance surpassed that of synthetic wastewater treatments. High turbidity removal was also observed in previous studies with PACl dosages of 600–1900 mg L^−1^ on MBR mixed liquor but without landfill leachate [20]. In our study, we achieved higher turbidity removal with lower dosages and co-treatment, with the lowest residual turbidity (0.1 NTU) being lower than the best results reported by [20], which ranged from 2.0 to 4.6 NTU using PACl and microfiltration membranes. Notably, studies involving Tanfloc and other organic coagulants in MBR systems have not typically evaluated turbidity.

Direct application of coagulants for high turbidity removal (>90%) was observed in all PACl applications [20,100,101]. However, the near-total turbidity removal achieved in this research (almost 100%) was more favorable than previous studies, which reported efficiencies ranging from 50% to 98%, even though smaller dosages (30 mg L^−1^) were used compared to the 1100–2000 mg L^−1^ commonly employed [101,107]. This suggests that co-treatment with an MBR system can achieve high turbidity removal without requiring high coagulant dosages and may offer superior results compared to treating landfill leachate alone.

High total suspended solids (TSS) removal, exceeding 99%, was achieved across all conditions analyzed. This finding is consistent with the literature on MBR systems and co-treatment with 2–5% LL *v*/*v* [85]. Therefore, the addition of coagulants did not negatively impact TSS removal, even under co-treatment conditions.

It should be noted that this study did not assess potential treatments for the sludge generated. However, the literature suggests that the reuse of the solid phase from treatment is a viable option. Sludge produced with aluminum-based coagulants can be repurposed for various applications, including reuse in coagulation/flocculation processes, adsorption of metals and phosphates, and agricultural soil enhancement. This is feasible due to the organic matter, macronutrients, and micronutrients present in the sludge. Nevertheless, it is crucial to exercise caution to prevent the solubilization of aluminum and the subsequent removal of phosphorus from the soil [108]. Similarly, sludge generated with tannin-based coagulants can be used to improve soil quality in agriculture due to its organic matter and nutrient content [109]. However, precautions must be taken to address the potential leaching of polluting compounds, such as phenols, that may be present in the coagulant. Additionally, specific attention is required regarding the pollutants found in landfill leachate, including heavy metals and recalcitrant compounds, which can be environmentally hazardous even in trace amounts. In such cases, a detailed characterization of the sludge prior to disposal is essential.

## 4. Conclusions

As anticipated, co-treatment with landfill leachate (LL) significantly intensified fouling, leading to increased irreversible and non-recoverable inorganic fouling. This intensified fouling impaired the previously elucidated fouling mechanisms and reduced filterability. The reduced ability to recover from fouling, combined with the increased presence of refractory, humic, and fulvic substances, further hindered the removal efficiency of organic foulants due to LL addition.

Regarding coagulants, both PACl and Tanfloc improved filterability. Among the analyzed parameters (flux decay mitigation, FI30 improvement, total fouling layer (FL) reduction, and total fouling resistance recovery (FRR) improvement), PACl proved effective. However, Tanfloc outperformed PACl in enhancing total FRR and mitigating irreversible and non-recoverable inorganic fouling. Both coagulants improved the removal efficiency of nearly all pollutants, with Tanfloc showing particularly notable results except for humic and fulvic substances, which require further investigation.

The impact of the coagulants on the accumulation of deposits varied. The addition of Tanfloc increased total fouling resistance, whereas PACl did not. Despite this, Tanfloc significantly improved fouling reversibility, as indicated by the lowest irreversible fouling resistance (R_i_) and the observed increases in reversible cake resistance (Rc) and organic resistance (R_o_). Additionally, Tanfloc resulted in the lowest irreversible fouling layer resistance (FL_ir_) among the co-treatment conditions. However, the reduction in Hermia model fitting accuracy suggested that the effect of Tanfloc on fouling mechanisms during the initial hour of filtration was less clear. Further studies are needed to confirm this effect.

In conclusion, while Tanfloc had a lesser impact on filterability compared to PACl, it demonstrated superior performance in pollutant removal efficiency and fouling reversibility. Its natural origin and reduced environmental impact further suggest its potential for enhancing mixed liquor ultrafiltration in wastewater and landfill leachate co-treatment. Both coagulants can improve the performance of urban wastewater treatment plants (WWTPs) by increasing organic matter removal and, in the case of PACl, phosphorus removal. Increasing flux recovery and reducing fouling irreversibility could potentially extend membrane lifespan. Nevertheless, Tanfloc showed the most favorable results in fouling mitigation and pollutant removal and may be a viable option for consideration in urban co-treatment settings.

## Figures and Tables

**Table 1:** Mixed liquor conditions on filtration experiments.

Sample	Mixed Liquor Affluent	Coagulant Dosage
1	Synthetic wastewater	0
2	Synthetic wastewater + LL 20 % (*v*/*v*)	0
3	Synthetic wastewater + LL 20 % (*v*/*v*)	30 mgPACl ^1^ L^−1^
4	Synthetic wastewater + LL 20 % (*v*/*v*)	30 mgTanfloc ^2^ L^−1^

^1^ The PACl dosage is shown in terms of active principle (Al_2_O_3_); ^2^ the Tanfloc dosage is shown in terms of solids.

**Table 2:** Co-treatment effect on flux loss and flux recovery rate.

Affluent	J_w0_ (LMH)	J (LMH)	FL (%)				FRR (%)		
			FL_t_	FL_r_	FL_io_	FL_ir_	FRR_T_	FRR_PC_	FRR_CC_
sWW	27.3	8.1	70	23	37	11	89	53	37
sWW + LL	35.8	10.7	70	31	18	21	79	61	18

**Table 3:** Co-treatment effect on RIS model.

Affluent	Resistance (10^12^ m^−1^)					
	Total (R_t_)	Membrane (R_m_)	Fouling (R_f_)	Fouling Type		
				Cake Layer (R_c_)	Internal Organic (R_io_)	Irrecoverable + Internal Inorganic (R_ir_)
sWW	3.92	1.32	2.60	1.41	1.03	0.16
		(34%)	(66%)	(54%)	(40%)	(6%)
sWW + LL	3.13	1.00	2.12	1.48	0.38	0.27
		(32%)	(68%)	(70%)	(18%)	(13%)

**Table 4:** Co-treatment effect on Hermia models.

Affluent	Linear Correlation Coefficient R^2^											
	0–30 min				30–60 min				60–120 min			
	CB	IB	SB	CF	CB	IB	SB	CF	CB	IB	SB	CF
sWW	0.976	0.993	0.993	0.996	0.720	0.834	0.834	0.831	0.909	0.932	0.932	0.938
sWW + LL	0.911	0.943	0.943	0.947	0.638	0.687	0.687	0.686	0.974	0.983	0.983	0.986

**Table 5:** Coagulants effect on flux loss and recovery.

Affluent	J_w0_ (LMH)	J (LMH)	FL (%)				FRR (%)		
			FL_t_	FL_r_	FL_io_	FL_ir_	FRR_T_	FRR_PC_	FRR_CC_
sWW + LL	35.8	10.7	70	31	18	21	79	61	18
sWW + LL + PACl	25.7	7.5	71	48	9	14	86	77	9
sWW + LL + Tanfloc	30.9	7.6	75	34	30	12	88	58	30

**Table 6:** Coagulants effect on RIS model.

Affluent	Resistance (10^12^ m^−1^)					
	Total (R_t_)	Membrane (R_m_)	Fouling (R_f_)	Fouling Type		
				Cake Layer (R_c_)	Internal Organic (R_io_)	Irrecoverable + Internal Inorganic (R_ir_)
sWW + LL	3.13	1.00	2.12	1.48	0.38	0.27
		(32%)	(68%)	(70%)	(18%)	(13%)
sWW + LL + PACl	4.24	1.40	2.85	2.43	0.19	0.25
		(33%)	(67%)	(86%)	(7%)	(8%)
sWW + LL + Tanfloc	4.28	1.16	3.11	2.28	0.65	0.19
		(27%)	(73%)	(73%)	(21%)	(6%)

**Table 7:** Coagulants effect on Hermia models.

Affluent	Linear Correlation Coefficient (R^2^)											
	0–30 min				30–60 min				60–120 min			
	CB	IB	SB	CF	CB	IB	SB	CF	CB	IB	SB	CF
sWW + LL	0.911	0.943	0.943	0.947	0.638	0.687	0.687	0.686	0.974	0.983	0.983	0.986
sWW + LL + PACl	0.945	0.974	0.974	0.979	0.998	0.951	0.951	0.955	0.971	0.932	0.932	0.924
sWW + LL + Tanfloc	0.362	0.105	0.105	0.101	0.564	0.601	0.601	0.598	0.977	1.000	1.000	1.000

## Data Availability

The data presented in this study are available on request from the corresponding author due to privacy reasons.

## Supplementary Materials

The following supporting information can be downloaded at: https://www.mdpi.com/article/10.3390/membranes14100212/s1, Table S1: MBR operational characteristics; Table S2: Synthetic wastewater composition; Table S3: Mixed liquor characteristics according to MBR affluent; Table S4: Landfill leachate characteristics; Table S5: Main characteristics of PACl and Tanfloc SG, according to manufacturer; Figure S1: Pi-lot-scale membrane bioreactor; Figure S2: Schematic draw of preliminary tests; Figure S3: Main results of the preliminary test; Figure S4: SMP and EPS extraction procedure (adapted from [53]); Figure S5: Visual effect of real color improvement on mixed liquor.
