# Supplementary material for: Landfill Leachate and Coagulants Addition Effects on Membrane Bioreactor Mixed Liquor: Filterability, Fouling, and Pollutant Removal
Source: Membranes (Basel). 2024 Oct 2;14(10):212. doi: 10.3390/membranes14100212 (PMC11509387; doi:10.3390/membranes14100212)
Supplement: Supplementary file 1 [file membranes-14-00212-s001.zip › membranes-3175445-supplementary.pdf]

Supplementary Material

Co-treatment and coagulants addition effects on MBR mixed liquor ultrafiltration: filterability, removal efficiency and fouling

Rodrigo Almeria Ragio[1], Ana Carolina Santana[1], and Eduardo Lucas Subtil[1,*]

Laboratory of Urban Wastewater Treatment and Water Reuse (LabTAUS), Engineering, Modelling and Ap-plied Social Sciences Center, Federal University of ABC, Santo André, SP, Brazil; edu-ardo.subtil@ufabc.edu.br.

Table S1. MBR operational characteristics according to reactor affluent

Function	Parameter	Unit	sWW		sWW + LL (20%)	
			Average	St. dev.	Average	St. dev.
Operational control (MBR)	Dissolved oxygen	mg L^{-1}	4,5	1,0	2,4	2,2
	Temperature	°C	24,0	1,3	25,0	2,9
	pH	-	6,6	0,3	6,4	0,6
	MLSS	mgL^{-1}	2,9	0,5	1,3	0,2

Table S2. Synthetic wastewater composition

Reagent	Theoretical concentration (mg.L^{-1})	Importance
Boric Acid (H_2BO_3)	0,45	Source of salts and micronutrients
Sugar	56	Organic carbon source
Sodium Bicarbonate ($NaHCO_3$)	416	Increase alkalinity (buffer)
Ammonium Chloride (NH_4Cl)	153	Ammoniacal nitrogen source (N-NH_3)
Calcium Chloride ($CaCl_2.2H_2O$)	4,5	Source of salts and micronutrients
Cobalt Chloride ($CoCl_2.6H_2O$)	0,45	Source of salts and micronutrients
Magnesium Chloride ($MgCl_2.6H_2O$)	7	Source of salts and micronutrients
Manganese Chloride II* ($MnCl_2.4H_2O$)	0,36	Source of salts and micronutrients
Sodium Chloride (NaCl)	250	Source of salts and micronutrients
Ferric Chloride ($FeCl_3$)	4,5	Source of salts and micronutrients
EDTA Disodium Salt	30	Solubilization of metals
Ethanol (C_2H_5OH)	19,93	Organic carbon source
Meat extract (Whey Protein)	166,4	Source of organic carbon and nitrogen
Monobasic Potassium Phosphate (KH_2PO_4)	36	Source of salts and micronutrients
Potassium Iodide (KI)	0,54	Source of salts and micronutrients
Sodium Molybdate ($NaMoO_4.2H_2O$)	0,18	Source of salts and micronutrients
Copper Sulfate ($CuSO_4.5H_2O$)	0,09	Source of salts and micronutrients
Zinc Sulphate ($ZnSO_4.7H_2O$)	0,36	Source of salts and micronutrients

Table S3. Mixed liquor characteristics according to MBR affluent

Parameter	Unit	Synthetic WW (sWW)		Synthetic WW + LL 20%	
		Average	Standard deviation	Average	Standard deviation
pH	-	6.4	0.2	7.2	0.3
Temperature	°C	23.0	0.2	22.6	0.7
MLSS	g L^{-1}	2.5	0.1	1.2	0.2

Table S4. Landfill leachate characteristics

Parameter	Value
Total Chemical Oxygen Demand (COD)	4,077 mgO_2 L^{-1}
Dissolved Organic Carbon (DOC)	473.3 mg L^{-1}
Total Dissolved Carbon (TDC)	1,730 mg L^{-1}
Dissolved Inorganic Carbon (DIC)	1,257 mg L^{-1}
Total Dissolved Nitrogen (TDN)	1,196 mg L^{-1}
Total Phosphorus (TP)	21 mg L^{-1}
Turbidity	9.6 NTU
Apparent color	980 uC
Real color	12,749 uC
Humic substances (Abs 254 nm)	12.7 cm^{-1}
Total Suspended Solids (TSS)	0.74 g L^{-1}

Table S5. Main characteristics of PACl and Tanfloc SG, according to manufacturer

Characteristc	PACl	Tanfloc SG
Description	prepolymerized coagulant of cationic charge	low molecular weight organic-cationic polymer of essentially vegetable
Aspect	viscous liquid	liquid
Color	slightly cloudy, amber	-
Density (g mL^{-1})	> 1.20	-
Solubidity in water	soluble in water	-
Total solids content (%)	16 – 18	30 – 34
pH	1.40 – 3.0	1.3 – 2.3
Viscosity (s, 25ºC)	-	< 50

a) Scheme

b) Photograph

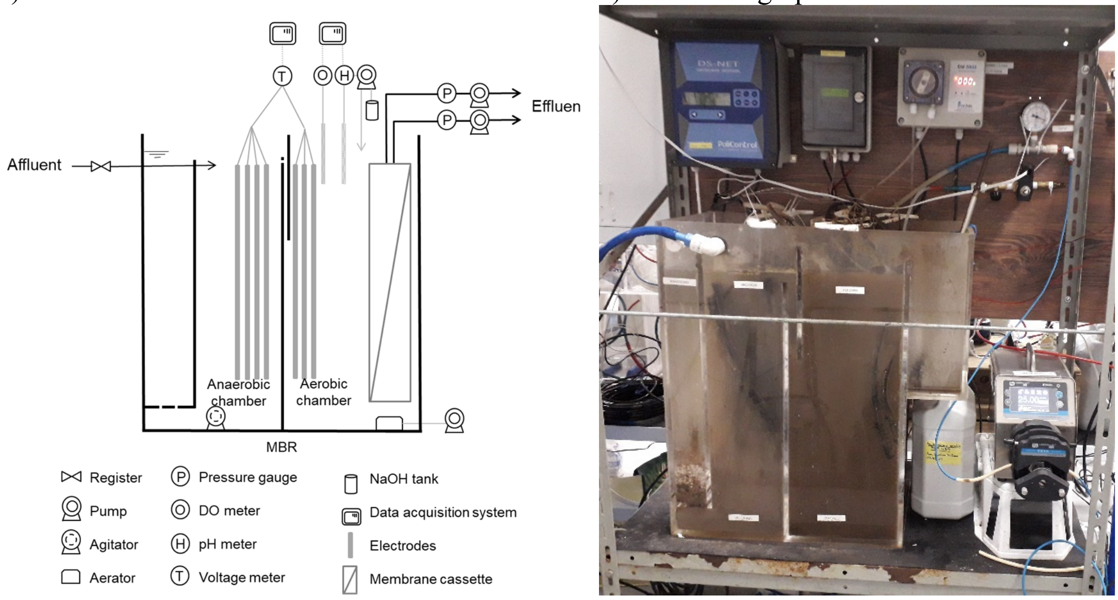

Figure S1. Pilot-scale membrane bioreactor

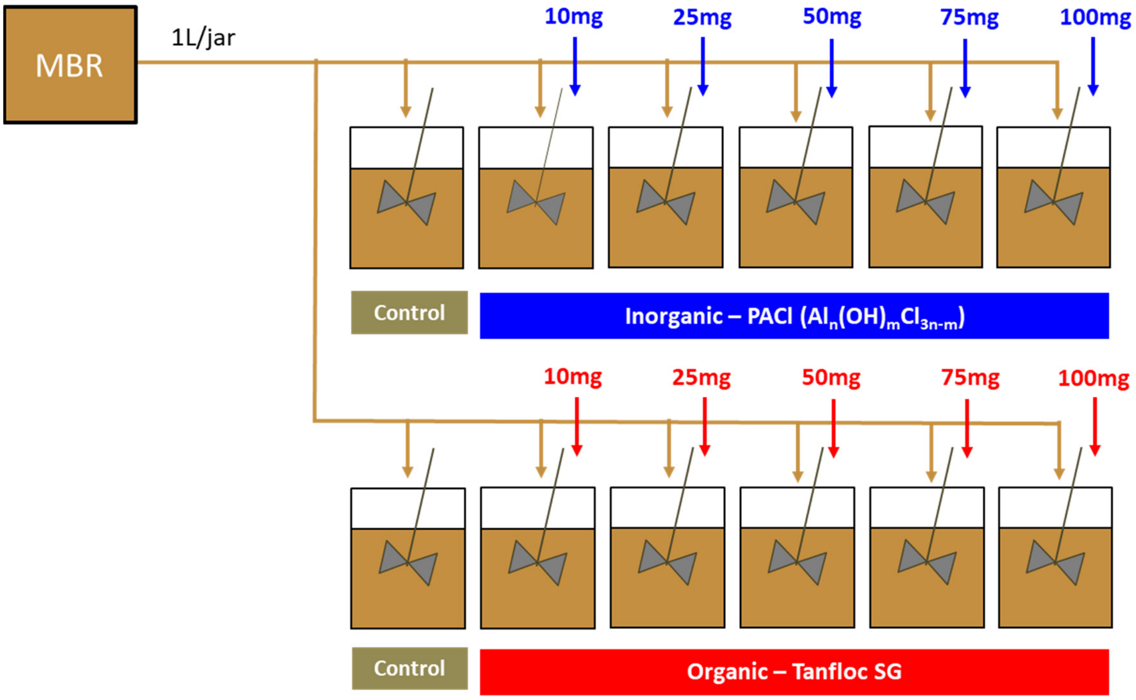

Figure S2. Schematic draw of preliminary tests

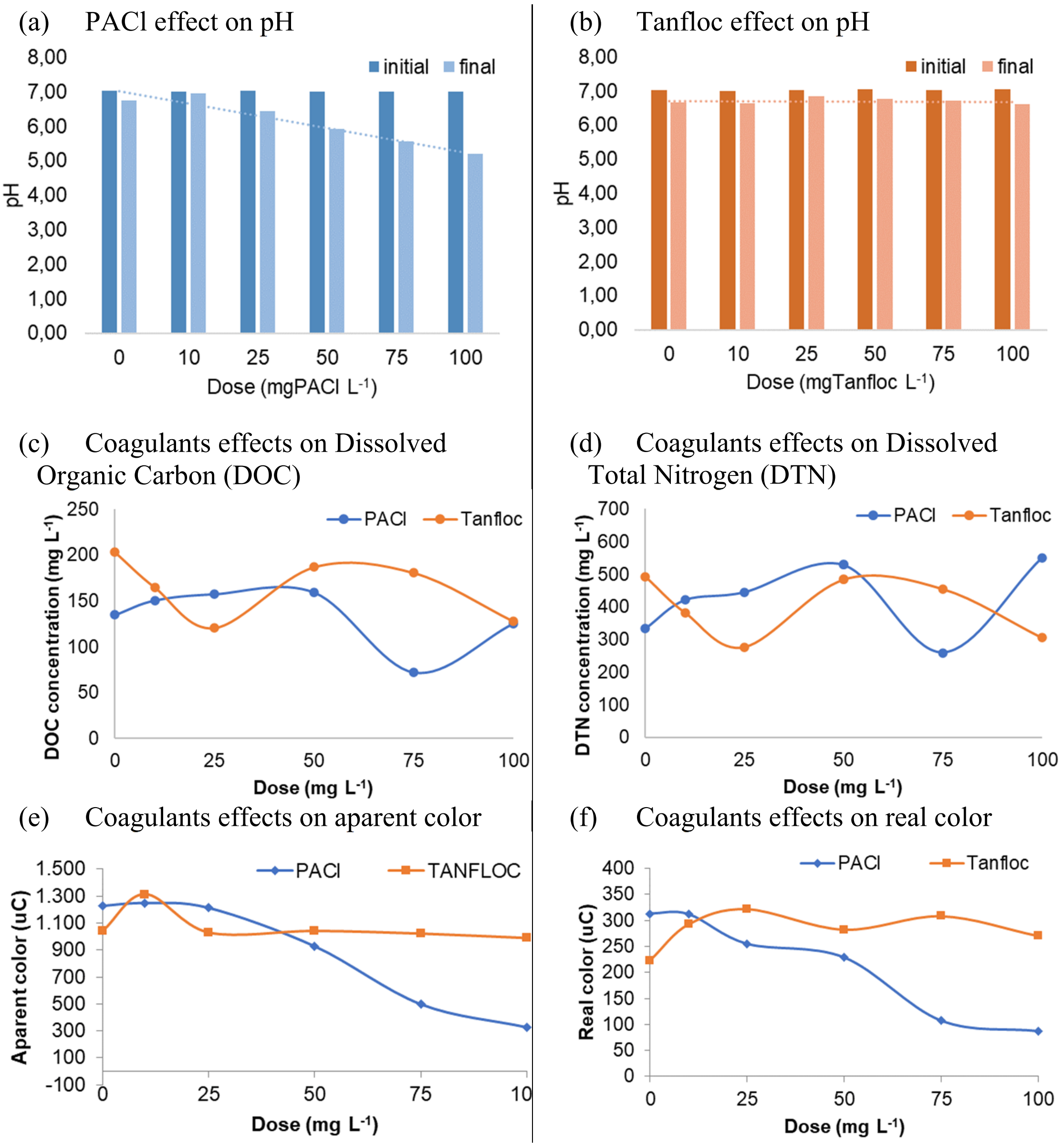

Figure S3. Main results of the preliminary test

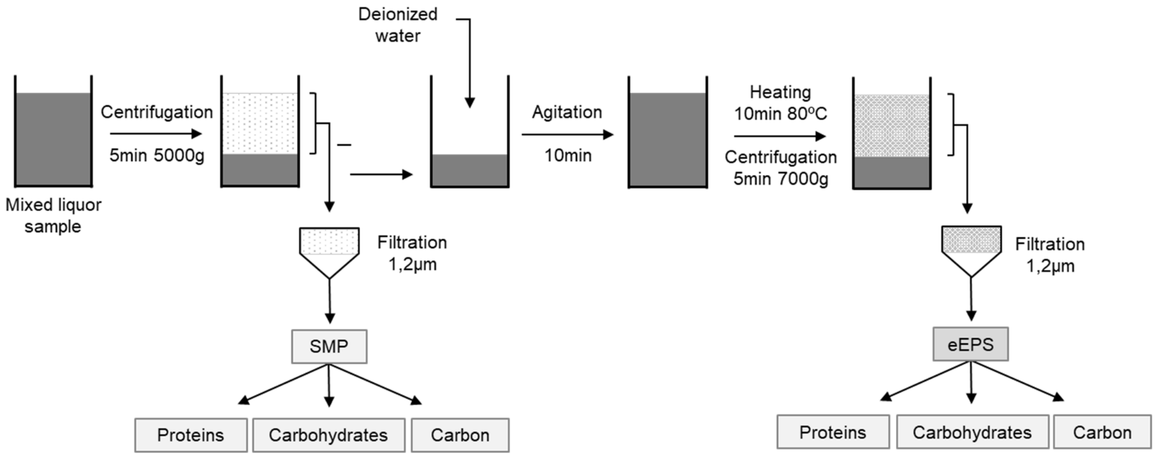

Figure S4. SMP and EPS extraction procedure. Adapted from [53].

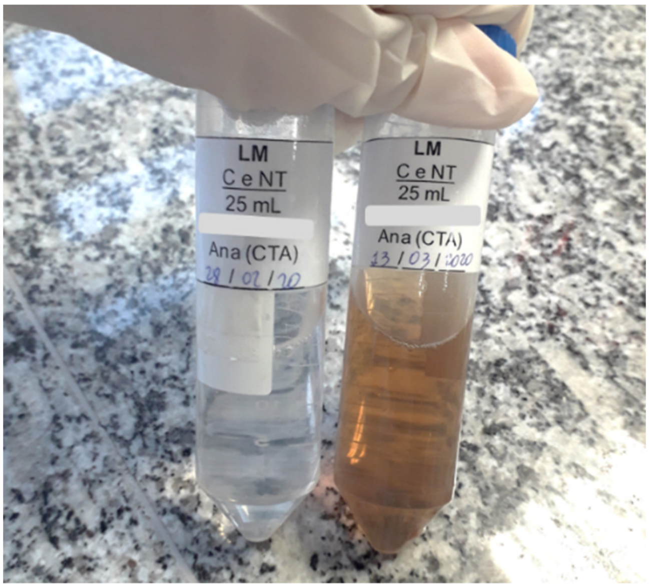

Figure S5. Visual effect of real color improvement on mixed liquor
